# A First-in-Human Randomized, Double-Blind, Placebo-Controlled, Single- and Multiple-Ascending Oral Dose Study of Novel Imidazolopiperazine KAF156 To Assess Its Safety, Tolerability, and Pharmacokinetics in Healthy Adult Volunteers

**DOI:** 10.1128/AAC.03478-14

**Published:** 2014-11

**Authors:** F. Joel Leong, Rong Zhao, Shuqi Zeng, Baldur Magnusson, Thierry T. Diagana, Peter Pertel

**Affiliations:** a Novartis Institute for Tropical Diseases, Singapore; b Novartis Institutes for BioMedical Research, Shanghai, People's Republic of China; c Novartis Pharma AG, Basel, Switzerland; d Novartis Institutes for BioMedical Research, Cambridge, Massachusetts, USA

## Abstract

KAF156 belongs to a new class of antimalarial, the imidazolopiperazines, and is currently in clinical development for the treatment of uncomplicated malaria. This first-in-human, single- and multiple-ascending-dose study in 70 healthy male volunteers determined the maximum oral dose of KAF156 tolerated by healthy adults and derived pharmacokinetic data (including preliminary food effect) to enable dose calculations for malaria patients. KAF156 was studied in single-dose cohorts (10 to 1,200 mg, including one 400-mg food effect cohort (4 to 10 subjects/cohort), and in multiple-dose cohorts (60 to 600 mg once daily for 3 days; 8 subjects/cohort). The follow-up period was 6 to 14 days after the last dose. KAF156 was tolerated, with self-limited mild to moderate gastrointestinal and neurological adverse events. In treated subjects after single doses, headache (*n* = 4; 11.1%), diarrhea (*n* = 3; 8.3%), dizziness (*n* = 3; 8.3%), and abdominal pain (*n* = 2; 5.6%) were the most common adverse events. Headache (*n* = 4; 16.7%), nausea (*n* = 3; 12.5%), upper respiratory tract infection (*n* = 3; 12.5%), and dizziness (*n* = 2; 8.3%) were the most common adverse events following multiple doses. KAF156 time to maximum concentration (*T*_max_) was between 1.0 and 6.0 h. Both the area under the concentration-time curve (AUC) and maximum concentration (*C*_max_) increased more than dose-proportionally in both single- and multiple-ascending-dose cohorts (terminal half-life, 42.5 to 70.7 h). There was no significant accumulation over 3-day repeated administration. The extent of absorption was not significantly affected by food at a single dose of 400 mg, while mean *C*_max_ decreased from 778 ng/ml to 627 ng/ml and *T*_max_ was delayed from a median of 3.0 h under fasting conditions to 6.0 h under fed conditions. Renal elimination is a minor route.

## INTRODUCTION

Malaria is caused mainly by two protozoan parasites, Plasmodium falciparum and Plasmodium vivax. The parasite infects hepatocytes and subsequently erythrocytes in the human host. It is the blood parasitemia and erythrocyte rupture which produce the acute flu-like symptoms (spiking fever, rigors), vascular compromise, and organ involvement, which results in complications. The existing mainstay of malaria therapy, artemisinin-containing combination therapies (ACT), has a questionable future given the recent documentation of artemisinin resistance in Southeast Asia (1–4). KAF156 (previously known as GNF156) belongs to a novel class of antimalarial, the imidazolopiperazines (5–7), and is active against a broad range of Plasmodium species, including drug-resistant parasites. Notably, the compound is parasiticidal against both asexual and sexual blood stages as well as the liver stages of the parasite, suggesting that KAF156 has the potential to prevent infection, treat acute disease, and reduce transmission (16). This sets KAF156 apart from other antimalarials, including the spiroindolone KAE609 (8–10). The mechanism of action is currently unknown, but drug resistance is mediated by the emergence of mutations in the cyclic amine resistance locus (PfCarl) (11), which encodes a protein of unknown function and contains several transmembrane domains (16).

The objectives of this first-in-human study were to assess the safety and tolerability of KAF156 in healthy adult volunteers after single and multiple oral dosing and to assess the pharmacokinetics after single dosing (including food effect) and multiple dosing.

## MATERIALS AND METHODS

### Study design.

This was a single-center, double-blind, randomized, placebo-controlled trial. It was conducted from 31 January 2012 to 2 June 2012 at the Q-Pharm Phase I Unit (Brisbane, Australia). This study comprised two parts.

Part 1 involved single-ascending-dose (SAD) cohorts with different doses of KAF156 (10 mg, 60 mg, 180 mg, 400 mg, 800 mg, and 1,200 mg) and one food effect cohort (400 mg) with 4 to 10 subjects (including placebo) in each cohort. It consisted of a maximum 27-day screening period, a baseline period (day −1), a treatment period (day 1), a follow-up period (days 2 to 15), and a single study completion evaluation on day 16.

Part 2 examined multiple-ascending-dose (MAD) cohorts with different doses (60 mg, 160 mg, 400 mg, and 600 mg once daily for 3 days), with 8 subjects in each cohort. It comprised a maximum 27-day screening period, a baseline period (day −1), a treatment period (days 1 to 3), a follow-up period (days 4 to 9), and a single study completion evaluation on day 10.

This study was conducted in compliance with the Declaration of Helsinki and the International Conference on Harmonization Guidelines for Good Clinical Practice. The final protocol, amendments, and informed consent documentation were reviewed and approved by the study center institutional review board. All subjects provided written, informed consent before participating in any study procedures.

### Subjects.

Healthy male and female subjects 18 to 55 years of age, weighing at least 50 kg, with a body mass index between 18 and 30 kg/m^2^ were eligible for the study. Good health was determined by medical history, physical examination, and laboratory tests at screening. Only females with no child-bearing potential were allowed to enroll, and all female subjects were required to have a negative pregnancy test result at screening and at the baseline.

Exclusion criteria included use of investigational drugs within 30 days or 5 half-lives of enrollment (whichever was greater); use of tobacco products within the previous 3 months; use of prescription medications or herbal supplements within the previous 4 weeks or over-the-counter medication (except for incidental acetaminophen), dietary supplements, or vitamins within 2 weeks; and blood donation or blood loss of >400 ml within the previous 8 weeks. Subjects with significant illness within the previous 2 weeks or a history of drug or alcohol abuse in the previous 12 months were also excluded.

### Safety assessment.

In both single- and multiple-dosing cohorts, safety assessments included physical examinations, electrocardiograms (ECGs), assessment of vital signs, and standard clinical laboratory evaluations (hematology, blood chemistry, and urinalysis), which occurred at least daily while patients were on site and during monitoring of adverse events and serious adverse events. Adverse events were recorded from time of first administration of study drug until study completion, while serious adverse events were recorded from time of consent until 30 days after stopping the trial.

In part 1 of the study, on day 1, following an overnight fast of at least 10 h, predose vital signs were assessed, and a predose blood sample was obtained for pharmacokinetics. A single dose of KAF156 or matching placebo was then administered. Subjects continued to fast for at least 4 h postdose. Subjects remained domiciled at the site. Following pharmacokinetic and safety assessment for up to 48 h postdose, subjects were discharged from the study site on day 3. They returned to the site on day 5 (96 h postdose), day 7 (144 h postdose), and day 9 (192 h postdose) for pharmacokinetic blood sampling as an ambulatory visit.

Part 2 (multiple dosing) began once adequate pharmacokinetic and safety data had been assessed from part 1 to enable estimation of an initial dose. Subjects reported to the unit on day −1 and remained in the unit until 48 h after the last dose. All doses were given following an overnight fast of at least 10 h. Subjects continued to fast for at least 4 h postdose on day 1 and day 3, with no breakfast provided. Breakfast was provided 2 h after dosing on day 2. Subjects remained on site until day 5 (48 h after the last dose) to facilitate collection of safety data and allow close observation. Study completion evaluation occurred on day 10 (168 h after the last dose).

At each visit, subjects underwent a standard physical examination, ensuring that any potential clinical symptoms or signs were detected. Subjects were also told to contact the study site between visits should they experience any problems. Additional assessments were performed in the event of an adverse event.

### Pharmacokinetic assessment.

Series of blood samples for measurement of KAF156 plasma levels were collected in EDTA tubes at predetermined time points: predose and 0.5, 1, 2, 3, 4, 6, 8, 12, 24, 48, 96, 144, 192, 264 (1,200-mg cohort only), and 360 (1,200-mg cohort only) h postdose following single-dose administration in part 1 and predose and 0.5, 1, 2, 3, 4, 6, 8, and 12 h postdose on day 1, predose on day 2, and predose and 0.5, 1, 2, 3, 4, 6, 8, 12, 24, 48, and 168 h after the last dose in part 2.

Urine samples were collected from the 400 (fasting period only)-, 800-, and 1,200-mg single-dose cohorts predose and at predetermined collection intervals: 0 to 12, 12 to 24, and 24 to 48 h postdose. Plasma and urine samples were analyzed for KAF156 using validated liquid chromatography-mass spectrometry/mass spectrometry (LC-MS/MS) methods with lower limits of quantification of 1 and 1,000 ng/ml, respectively.

The pharmacokinetic parameters were calculated from the KAF156 plasma concentration–actual-time data for subjects taking active treatment by standard noncompartmental methods using WinNonlin Phoenix (version 6.2). The pharmacokinetic parameters include area under the concentration-time curve from time zero to 24 h postdose (AUC_0–24_), AUC from time zero to the time of the last quantifiable concentration (mass × time/volume) (AUC_last_), AUC from time zero extrapolated to infinity (AUC_∞_), maximum observed concentration (*C*_max_), time of occurrence of *C*_max_ (*T*_max_), elimination half-life (*t*_1/2_), apparent oral clearance (CL/*F*), apparent volume of distribution (*V_z_*/*F*), and accumulation ratio (R_acc_), which was defined as the ratio of AUC_0–24_ (day 3) to AUC_0–24_ (day 1). In addition, urinary excretion of KAF156 during the collection interval (Ae0-t) and renal clearance (CL_r_) were estimated from the urine concentration and volume-time interval data collected from subjects receiving 400, 800, and 1,200 mg during part 1. Subjects for whom selected pharmacokinetic parameters could not be adequately characterized were excluded from the calculation of overall means and statistics for those parameters.

### Statistical methods.

Adverse events were summarized by counts. Laboratory tests, vital signs, and electrocardiogram (ECG) parameters were summarized by descriptive statistics by part, cohort, and visit at each time point.

Pharmacokinetic parameters were summarized using arithmetic means and standard deviations except for *T*_max_, for which median values and ranges are reported. To test dose proportionality, the primary pharmacokinetic parameters were analyzed using a regression model on the log-transformed pharmacokinetic parameters (*C*_max_, AUC_0–24_, and/or AUC_∞_) versus log-transformed dose, assessing dose proportionality with a lack-of-fit test. The analyses were done separately for single- and multiple-dose cohorts and separately on days 1 and 3 for the multiple-dose cohorts. To assess food effect, data from the same subjects under fed and fasting conditions were pooled, and AUC_∞_, AUC_last_, and *C*_max_ were analyzed by mixed-effect model analysis of variance with the fasting or fed condition as a fixed effect and a compound symmetric covariance within each subject.

## RESULTS

### Subject demographics.

A total of 70 subjects (38 subjects in part 1 and 32 subjects in part 2) were enrolled in the study, and 67 subjects completed the study, among whom 29 and 24 subjects received KAF156 in parts 1 and 2, respectively. All enrolled subjects were males. Demographic data are summarized in Table 1. All subjects with evaluable data were included in safety and pharmacokinetics (PK) analyses.

**TABLE 1:** Demographic summary

Characteristic	Part 1 (single dosing)			Part 2 (multiple dosing)		
	KAF156 (*n* = 29)	Placebo (*n* = 9)	Total (*n* = 38)	KAF156 (*n* = 24)	Placebo (*n* = 8)	Total (*n* = 32)
Mean age (yr)^*a*^	24.7 (18–44)	30.3 (21–46)	26.0 (18–46)	26.4 (20–50)	25.8 (21–31)	26.3 (20–50)
No. (%) of males	29 (100)	9 (100)	38 (100)	24 (100)	8 (100)	32 (100)
No. (%) of:						
Caucasians	23 (79.3)	8 (88.9)	31 (81.6)	22 (91.7)	8 (100)	30 (93.8)
Blacks	1 (3.4)	0 (0.0)	1 (2.6)	0 (0.0)	0 (0.0)	0 (0.0)
Asians	5 (17.2)	1 (11.1)	6 (15.8)	0 (0.0)	1 (4.2)	1 (3.1)
Pacific Islanders	0 (0.0)	0 (0.0)	0 (0.0)	0 (0.0)	1 (4.2)	1 (3.1)
Wt (kg)^*a*^	78.8 (59–101)	76.9 (62–86)	78.4 (59–101)	80.2 (60–102)	79.9 (64–88)	80.1 (60–102)
Ht (cm)^*a*^	177.86 (166–192)	180.89 (172–189)	178.58 (166–192)	181.25 (169–205)	183.88 (173–189)	181.91 (169–205)

a Data are means (ranges).

### Safety and tolerability.

All 70 subjects were included in the safety analysis (subjects who returned to be included in the fed cohort were counted twice). There were no deaths or serious adverse events. The overall incidence of adverse events was similar in treated subjects and placebo subjects in the single dose part of the study but was higher in treated subjects than placebo subjects in the multiple-dose part of the study (Tables 2 and 3).

**TABLE 2:** Incidence of adverse events in part 1 (single dosing)

Adverse event	No. (%) receiving:						
	10 mg (*n* = 3)	60 mg (*n* = 3)	180 mg (*n* = 3)	400 mg (*n* = 8)	800 mg (*n* = 6)	1,200 mg (*n* = 6)	Placebo, pooled (*n* = 9)
Any adverse event(s)	0	2 (66.7)	2 (66.7)	2 (25.0)	2 (33.3)	5 (83.3)	4 (44.4)
Any drug-related adverse event(s)	0	0	0	0	0	3 (50.0)	2 (22.2)
Discontinuation due to an adverse event	0	0	0	0	0	1 (16.7)	0
Diarrhea	0	0	0	0	1 (16.7)	2 (33.3)	1 (11.1)
Abdominal pain	0	0	0	1	0	1 (16.7)	0
Nausea	0	0	0	0	1 (16.7)	0	0
Gastritis	0	0	0	1 (12.5)	0	0	0
Vomiting	0	0	0	0	0	1 (16.7)	1 (11.1)
Headache	0	2 (66.7)	0	1 (12.5)	0	1 (16.7)	1 (11.1)
Dizziness	0	0	0	0	1 (16.7)	2 (33.3)	0
Neuralgia	0	1 (33.3)	0	0	0	0	0
Presyncope	0	0	0	0	0	1 (16.7)	0
Contusion	0	0	0	0	0	1 (16.7)	1 (11.1)
Joint injury	0	0	1 (33.3)	0	0	0	0
Upper respiratory tract infection	0	0	0	1 (12.5)	0	0	0
Rhinitis	0	0	0	0	0	0	1 (11.1)
Pain in extremity	0	0	0	0	0	1 (16.7)	1 (11.1)
Hematuria	0	0	1 (33.3)	0	0	0	0
Oropharyngeal pain	0	0	0	0	0	1 (16.7)	0

**TABLE 3:** Incidence of adverse events in part 2 (multiple dosing)

Adverse event	No. (%) receiving:					
	60 mg (*n* = 6)	160 mg (*n* = 6)	400 mg (*n* = 6)	600 mg (*n* = 6)	KAF156, pooled (*n* = 24)	Placebo, pooled (*n* = 8)
Any adverse event(s)	5 (83.3)	2 (33.3)	4 (66.7)	2 (33.3)	13 (54.2)	1 (12.5)
Any drug-related adverse event(s)	3 (50.0)	0	0	1 (16.7)	4 (16.7)	0
Discontinuation due to an adverse event	0	0	0	0	0	0
Headache	3 (50.0)	1 (16.7)	0	0	4 (16.7)	0
Dizziness	1 (16.7)	0	1 (16.7)	0	2 (8.3)	0
Nausea	0	0	1 (16.7)	2 (33.3)	3 (12.5)	0
Upper respiratory tract infection	1 (16.7)	0	2 (33.3)	0	3 (12.5)	1 (12.5)
Excoriation	0	1 (16.7)	0	0	1 (4.2)	0
Laceration	1 (16.7)	0	0	0	1 (4.2)	0
Increased alanine aminotransferase	0	0	1 (16.7)	0	1 (4.2)	0

There was no dose-related trend with regard to frequency of adverse events among the KAF156-treated subjects except for gastrointestinal events, which collectively showed an increase from 12.5% to 50% in single-dose cohorts from 400 mg to 1,200 mg and 16.7% to 33.3% in multiple-dose cohorts from 400 to 600 mg (daily dosing for 3 days). However, the individual rates of nausea, vomiting, and diarrhea were similar in the pooled KAF156 and placebo groups, and there was no clear dose response for these events. Abdominal pain was noted in only two subjects receiving KAF156, with no clear dose response. There may be a dose-related trend for nausea after multiple dosing (1 subject receiving 400 mg daily for 3 days and 2 subjects receiving 600 mg daily for 3 days).

After single doses, 44.8% (13/29) and 44.4% (4/9) of subjects administered KAF156 and placebo, respectively, developed one or more adverse events. In part 1, the 9 subjects who presented for the 400-mg dose under fasting conditions then returned for the 400-mg dose under fed conditions. Two of 7 subjects who received KAF156 in the fed cohort developed 5 adverse events (abdominal pain, excoriation, eye injury, laceration, and peripheral edema). Both subjects who received placebo developed headaches. In the fasting and fed cohorts, the most commonly reported adverse events in the KAF156-treated subjects were headache (*n* = 4; 11.1%), diarrhea (*n* = 3; 8.3%), dizziness (*n* = 3; 8.3%), and abdominal pain (*n* = 2; 5.6%). In the placebo-treated subjects, only headache was reported in more than one subject (*n* = 2; 22.2%). Five events were reported in subjects administered KAF156 or placebo, including headache (11.1% versus 22.2%, respectively), diarrhea (8.3% versus 11.1%), vomiting (2.8% versus 11.1%), contusion (2.8% versus 11.1%), and pain in an extremity (2.8% versus 11.1%).

All adverse events were rated grade 1 or 2 according to *Common Terminology Criteria for Adverse Events (CTCAE)* (12) in severity and resolved spontaneously. Overall, the grade 2 adverse events (6/36 subjects; 16.7%) were as follows for the pooled KAF156 single-dose groups: gastritis, vomiting, diarrhea, contusion, headache, dizziness, laceration, neuralgia, presyncope, and eye injury. In the pooled placebo single-dose groups, there were 2 subjects (2/11, 18.2%) reporting 3 grade 2 adverse events (diarrhea, vomiting, and rhinitis).

One subject in the KAF156 1,200-mg single-dose group discontinued the study due to an adverse event which occurred while in the bathroom on day 1, approximately 20 min after his dose. It was recorded as a vasovagal episode and described by the subject as comprising dizziness and lightheadedness with a small amount of vomiting. Following this episode, the subject vomited a larger volume (200 ml) at 2 h postdose. The subject was assessed by the investigator and withdrawn from the study on the same day. Pharmacological intervention was not required. No other symptoms were reported. The subject was stable on discharge, nearly 7 h after dosing. No significant abnormalities were detected on examination of blood taken during this period. As he was withdrawn from the study, this subject was excluded from pharmacokinetic parameter calculations.

Two other subjects experienced diarrhea, with one also developing abdominal pain, on day 1, within 2 to 3 h of their 1,200-mg dose. No treatment was required and the symptoms resolved. To help facilitate dose selection for part 2, the dosing regimens for these subjects were unblinded; all three had received KAF156. One subject who had received placebo in this cohort reported a single episode of diarrhea.

After multiple doses, 54.2% (13/24) and 12.5% (1/8) of subjects administered KAF156 and placebo, respectively, developed one or more adverse events. The most commonly reported adverse events following multiple dosing in the KAF156-treated subjects were headache (*n* = 4; 16.7%), nausea (*n* = 3; 12.5%), upper respiratory tract infection (*n* = 3; 12.5%), and dizziness (*n* = 2; 8.3%). Upper respiratory tract infection was reported in both treated (12.5%) and placebo subjects (12.5%). In the multiple-dose groups, only laceration (one subject who received KAF156) was rated as grade 2 in severity.

Overall, abdominal pain, diarrhea, vomiting, headache, and nausea were adverse events that were suspected by the investigator to be related to the study medication in at least one instance. Headache, diarrhea, and vomiting were also reported in placebo subjects. Although there were sporadic hematology, chemistry, or urinalysis parameters outside normal ranges among subjects receiving KAF156 (as well as the placebo group), none were considered clinically significant or drug related. Mean values for most of the hematology and blood chemistry parameters remained within the normal range from baseline to the end of the study, and there were no significant differences between placebo and control mean values.

One subject (receiving 400 mg KAF156 daily for 3 days) had an elevated alkaline phosphatase (ALT) level, ranging from 46 to 102 U/liter. Based on the laboratory reference range of 5 to 40 U/liter, this is no more than a CTCAE grade 1 abnormality. Of note is that the ALT level was slightly above the upper limit at screening (54 U/liter) and at baseline (59 U/liter). The last reading was 46 U/liter with a decreasing trend. Other liver enzymes were within reference range during this period. Bilirubin was also normal. No other abnormal values were reported as an adverse event or had an effect on subject disposition. There were no CTCAE grade 2 or higher abnormalities.

Most vital sign measurements were within normal ranges. There were no recorded adverse events related to vital signs and no trend in mean or median values indicating subclinical abnormality. ECG measurement did not reveal any significant QTc prolongations nor any abnormalities determined to be clinically significant.

### Pharmacokinetic assessment.

**FIG 1:**
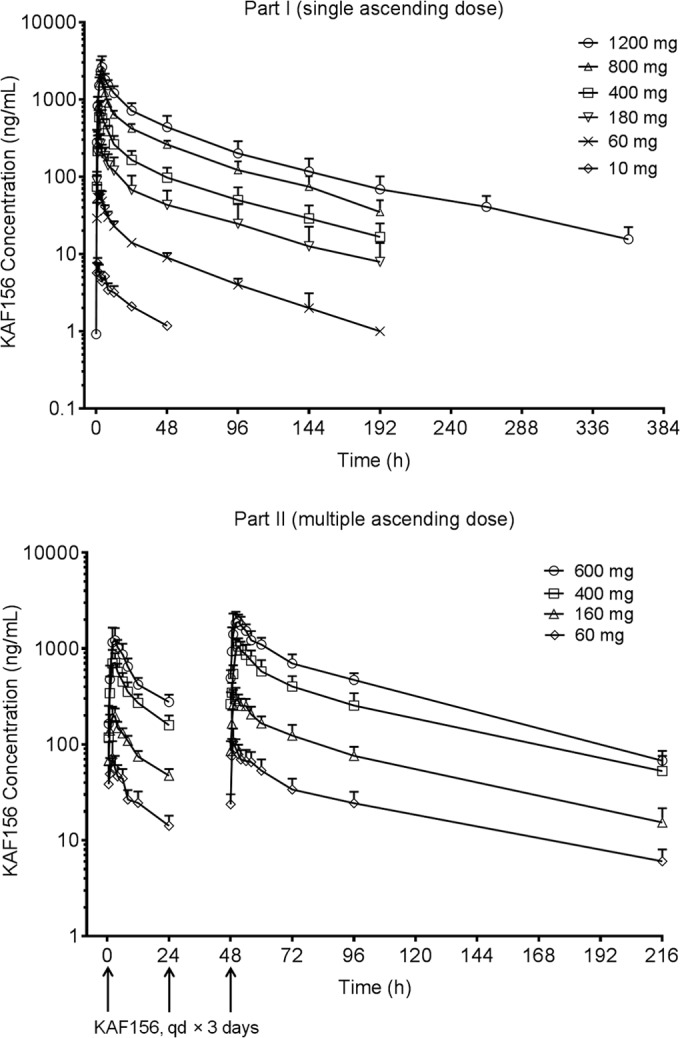
Arithmetic mean plasma concentration-time profiles of KAF156 following oral administration of single and multiple ascending doses.

The mean plasma concentration-time profiles of KAF156 under fasting conditions are presented in Fig. 1. The pharmacokinetic parameters are summarized in Table 4 (part 1, single ascending dose) and Table 5 (part 2, multiple ascending dose).

**TABLE 4:** Summary of pharmacokinetic parameters of KAF156 following oral administration of single ascending doses (part 1)^*a*^

Dose (mg)	AUC_0–24_ (ng · h/ml)	AUC_last_ (ng · h/ml)	AUC_inf_ (ng · h/ml)	*C*_max_ (ng/ml)	*T*_max_ (h)	*t*_1/2_ (h)	CL/*F* (liters/h)	*V_z_*/*F* (liters)
10 (*n* = 3)	83.0 ± 14.6	123 ± 16.2		7.6 ± 1.29	1.00 (1.00, 1.00)			
60 (*n* = 3)	679 ± 102	1,560 ± 205	1,710 ± 216	67.6 ± 9.79	1.02 (1.02, 4.00)	47.1 ± 4.35	35.6 ± 4.81	2,410 ± 222
180 (*n* = 3)	3,180 ± 1,240	7,550 ± 4,170	8,210 ± 4,830	275 ± 82.9	2.00 (2.00, 3.00)	51.7 ± 9.14	26.8 ± 12.6	1,890 ± 640
400 (*n* = 8)	7,570 ± 1,380	17,200 ± 4,890	18,700 ± 5,650	796 ± 177	3.00 (2.00, 6.00)	55.6 ± 10.2	23.8 ± 9.52	1,820 ± 440
800 (*n* = 6)	19,500 ± 2,340	43,700 ± 6,080	46,900 ± 6,870	2,050 ± 328	3.00 (2.00, 4.00)	48.0 ± 5.83	17.4 ± 2.51	1,200 ± 176
1,200 (*n* = 6)	31,200 ± 7,200	78,800 ± 24,800	80,300 ± 25,400	2,720 ± 949	4.00 (3.00, 6.00)	70.7 ± 9.78	16.0 ± 4.24	1,610 ± 381

a All values are means ± standard deviations except *T*_max_, which are medians (ranges). PK parameters that could not be adequately characterized were excluded.

**TABLE 5:** Summary of pharmacokinetic parameters of KAF156 following oral administration of multiple ascending doses (part 2)^*a*^

Dose (mg)	AUC_0–24_ (ng · h/ml)		*C*_max_ (ng/ml)		*T*_max_ (h)		*t*_1/2_ (h)		CL_ss_/*F* (liters/h)		*V_z_/F* (liters)		R_acc_
	Day 1	Day 3	Day 1	Day 3	Day 1	Day 3	Day 1	Day 3	Day 1	Day 3	Day 1	Day 3	
10	704 ± 196	1,360 ± 373	78.7 ± 29.1	112 ± 34.8	1.5 (0.5, 2.0)	1.0 (1.0, 3.0)		59.8 ± 4.8		47.8 ± 15.9		4,070 ± 1,170	1.93 ± 0.027
160	2,220 ± 281	4,460 ± 850	213 ± 46.6	341 ± 80.6	2.5 (2.0, 3.0)	2.0 (1.0, 4.0)		51.7 ± 5.1		37.0 ± 6.97		2,780 ± 688	2.01 ± 0.279
400	7,680 ± 1,830	15,100 ± 4,060	762 ± 224	1,100 ± 252	2.5 (2.0, 3.0)	2.5 (2.0, 4.0)		50.7 ± 2.9		28.3 ± 8.61		2,050 ± 497	1.96 ± 0.159
600	13,100 ± 2,360	27,800 ± 5,500	1,380 ± 308	2,210 ± 629	2.5 (2.0, 6.0)	3.0 (1.0, 4.0)		42.5 ± 2.1		22.5 ± 5.60		1,380 ± 340	2.12 ± 0.130

a All values are means ± standard deviations except *T*_max_, which are medians (ranges). *n* = 6 for each group.

KAF156 was absorbed rapidly, with a *T*_max_ ranging from 1.0 to 6.0 h following single (10 to 1,200 mg) and multiple (60 to 600 mg) ascending oral doses. Following single oral administration of KAF156 (10 to 1,200 mg), mean *C*_max_ (in the range of 7.6 to 2,720 ng/ml) and AUC_last_ (in the range of 123 to 78,800 ng · h/ml) increased in a dose-dependent but more than dose-proportional manner over the 10- to 1,200-mg dose range. There was a statistically significant deviation from dose proportionality for AUC_last_ (slope, 1.33; 90% confidence interval [CI], 1.27 to 1.39), AUC_∞_ (slope, 1.27; 90% CI, 1.16 to 1.38), and *C*_max_ (slope, 1.25; 90% CI, 1.20 to 1.30) over the dose range of 10 to 1,200 mg of KAF156. The mean elimination half-life ranged from 47.1 to 55.6 h for all <1,200-mg-dose cohorts, and a mean half-life of 70.7 h was observed for the 1,200-mg-dose cohort.

Following three-times-daily oral administration of KAF156 (60 to 600 mg), mean *C*_max_ and AUC_0–24_ on day 1 were in the ranges of 78.7 to 1,380 ng/ml and 704 to 13,100 ng · h/ml, respectively. The corresponding values for day 3 were 112 to 2,210 ng/ml and 1,360 to 27,800 ng · h/ml, respectively. The elimination half-life was not calculated on day 1 because of the long half-life and once-daily dosing of KAF156. The elimination half-life was in the range of 42.5 to 59.8 h over the 60- to 600-mg dose range. The mean accumulation ratio (R_acc_) ranged from 1.93 to 2.12, indicating no significant accumulation of KAF156 in systemic circulation. Following multiple daily administrations of KAF156, there was a statistically significant deviation from dose proportionality for AUC_last_ (slope, 1.28; 90% CI, 1.17 to 1.38), AUC_∞_ (slope, 1.25; 90% CI, 1.14 to 1.36), and *C*_max_ (slope, 1.29; 90% CI, 1.17 to 1.40) on day 3 over the dose range of 60 to 600 mg. The systemic exposure (*C*_max_ and AUC) increased in a more-than-dose-proportional manner.

Preliminary food effect (with a standard FDA-recommended high-fat breakfast) on pharmacokinetics of KAF156 was evaluated with a 400-mg single dose. The extent of absorption was not affected significantly by food intake, as the geometric mean ratios in fed versus fasting groups of AUC_0–24_ and AUC_∞_ were 0.961 (90% CI, 0.76 to 1.22) and 0.955 (90% CI, 0.74 to 1.24), respectively, while geometric mean *C*_max_ decreased from 778 ng/ml to 627 ng/ml (∼19% decrease, geometric mean ratio for fed versus fasting subjects of 0.806 [90% CI, 0.65 to 1.01]) after food intake. *T*_max_ was delayed from a median of 3.0 (range, 2.0 to 6.0) hours under fasting conditions to 6.0 (range, 2.0 to 6.0) hours under fed conditions. There was no major impact of food on apparent clearance (CL/*F*) (23.9 ± 4.94 liters/h for fed patients versus 23.8 ± 9.52 liters/h for fasting patients) or apparent volume of distribution (*V_z_*/*F*) (1,860 ± 235 liters for fed patients versus 1,820 ± 440 liters for fasting patients).

Urinary excretion of KAF156 was evaluated at single doses of 400, 800, and 1,200 mg. The cumulative amount of KAF156 recovered from the urine samples collected over a 48-h interval (about one half-life) was generally less than 5% of the administered dose for all subjects. The calculated mean renal clearance (CL_r_) was approximately 1.0 liters/h (1.03 ± 0.411, 1.07 ± 0.169, and 1.04 ± 0.297 liters/h at 400, 800, and 1,200 mg, respectively), suggesting a minor renal elimination route for KAF156, compared with the systemic clearance of 16.0 to 23.8 liters/h.

## DISCUSSION

New antimalarial agents continue to be needed because of the development of resistance to nearly every agent in current use (13–15). The ideal combination regimen to treat malaria is one that can be administered as a single dose or at least that requires administration for no more than 3 days. The regimen should provide rapid symptomatic relief, avoid prolonged subtherapeutic exposure to either agent in the combination, be able to treat all patients at risk (including neonates and pregnant women), and be well tolerated. KAF156 was generally well tolerated, with all adverse events being self-limited and assessed as mild to moderate (grades 1 and 2) in severity. There were no deaths or serious adverse events. The most common adverse events were diarrhea, nausea, abdominal discomfort, dizziness, and headache. No obvious dose-related trends in the frequency of specific adverse events were noted. However, gastrointestinal events developed more frequently with higher doses of KAF156 administered as single or multiple doses. Only one subject did not complete the study because of an adverse event; no subject stopped the study drug because of an adverse event.

Following single- or multiple-oral-dose administration, KAF156 was absorbed rapidly, with peak exposure being reached in 1.0 to 6.0 h. KAF156 demonstrated nonlinear pharmacokinetics in the dose range tested. The mechanism is unknown. However, the data suggest the potential for metabolic enzyme saturation and possible saturation of first-pass metabolism, which may increase the systemically available fraction (*F*) of KAF156 and decrease the apparent clearance (CL/*F*) and volume of distribution (*V_z_*/*F*). The terminal half-life is around 50 h at doses up to 800 mg, which would support once-daily dosing. KAF156 is a first-in-class compound with a novel mechanism of action that potentially acts on both liver and blood stages of the malaria parasite. Little information about the PK/pharmacodynamic (PD) relationship is available. Based on the pharmacokinetic results in this study, a daily dose of 160 mg for 3 days is able to achieve an efficacious exposure AUC_0–24_ of at least 1,800 ng · h/ml, which is equivalent to the efficacious dose in mice, while a daily dose of 400 mg for 3 days or a single dose of 800 mg is able to maintain a total plasma concentration greater than the MIC for 6 days. Overall, the KAF156 pharmacokinetic profile supports a single- or multiple-dose regimen for treating malarial patients. However, a decision regarding dosing regimen will require further assessment of pharmacokinetic and pharmacodynamic effect in malaria patients.

In a previous study (16), mice infected with Plasmodium berghei did not develop malaria when given 10 mg/kg of KAF156 2 h before infection. In cultures, early-stage gametocytes treated with KAF156 on days 8 to 12 following induction of gametocytogenesis showed a significant dose-dependent reduction in the total number of stage V gametocytes with the capacity to exflagellate. Mosquitoes fed 5 nM KAF156 failed to yield any oocysts. Additionally, P. berghei-infected mice treated with a single oral dose of KAF156 at 100 mg/kg were found not to transmit infection to Anopheles mosquitoes feeding on their blood (16). These findings foreshadow the potential application of KAF156 as a causal prophylactic antimalarial and also as a gametocidal agent for the control of malaria transmission in endemic populations.

KAF156 was well tolerated, with self-limited mild to moderate (CTCAE grade 1 to 2) gastrointestinal and neurological adverse events (diarrhea, nausea, abdominal discomfort, dizziness, and headache). In healthy male adult volunteers, KAF156 demonstrated PK dose overproportionality in both single and daily dosing for 3 days, with minimal food effect with respect to the extent of oral absorption. There were no findings that would preclude giving this compound to malaria patients, although the dose-related trend for gastrointestinal events is potentially of concern if high dosing is required. A malaria patient study using a 3-day regimen of 400 mg once daily began in 2013. That study was ongoing at the time writing of this article.
